# Recognizing Racism in Medicine: A Student-Organized and Community-Engaged Health Professional Conference

**DOI:** 10.1089/heq.2019.0015

**Published:** 2019-08-12

**Authors:** Ademide A. Adelekun, Sourik Beltrán, Julia Carney, Elle Lett, Whitney U. Orji, Emily Rider-Longmaid, Daniel C. Stokes, Stephanie Teeple, Jaya Aysola

**Affiliations:** ^1^ Perelman School of Medicine, University of Pennsylvania, Philadelphia, Pennsylvania.; ^2^ Department of Medical Ethics and Health Policy, University of Pennsylvania, Philadelphia, Pennsylvania.; ^3^ Leonard Davis Institute of Health Economics, University of Pennsylvania, Philadelphia, Pennsylvania.; ^4^ Department of Biostatistics, Epidemiology, and Informatics, University of Pennsylvania, Philadelphia, Pennsylvania.; ^5^ Division of General Internal Medicine, Department of Medicine, University of Pennsylvania, Philadelphia, Pennsylvania.; ^6^ Office of Inclusion and Diversity, Perelman School of Medicine, University of Pennsylvania, Philadelphia, PA.

**Keywords:** racism, medical education, gender, diversity, sexual orientation, interprofessional collaboration

## Abstract

**Purpose:** This piece details the evaluation and implementation of a student-led educational intervention designed to train health professionals on the impact of racism in health care and provide tools to mitigate it. In addition, this conference, cosponsored by medical, nursing, and social work training programs, facilitates development of networks of providers with the knowledge and skills to recognize and address racism in health care.

**Methods:** The conference included 2 keynote speakers, an interprofessional panel, and 15 workshops. Participants (*n*=220) were asked to complete a survey assessing perceptions of conference content and impact. We compared responses pre- and postconference using Wilcoxon signed-rank tests.

**Results:** Of the survey respondents (*n*=44), 45.5% were medical students, 13.6% nursing students, and 9% social work students; 65.9% self-identified as a race/ethnicity other than non-Hispanic white; and 63.6% self-identified as female. We found that 47.7% respondents reported they were more comfortable discussing how racism affects health (*p*<0.001), 36.4% had better understanding of the impact of racism on an individual's health (*p*<0.001), and 54.5% felt more connected to other health professionals working to recognize and address racism in medicine (*p*<0.001).

**Conclusion:** These findings suggest that a student-organized conference could potentially be an effective strategy in addressing a critical gap in racism training for health care professionals.

## Introduction

The role of structural racism on the health of minority communities is well documented in the current literature.^1–3^ Structural racism, or the totality of ways in which societies foster racial discrimination through mutually reinforcing inequitable systems (Bailey et al., p. 1453), observable in housing, education, employment, criminal justice, and health care, contributes to significantly higher morbidity and mortality among racial minorities.^4^ Moreover, research has also demonstrated that health care providers exacerbate these disparities by providing racially biased treatment.^5^

In 2000, the Liaison Committee on Medical Education (LCME) mandated that medical schools prepare their students to understand health disparities and to treat a diverse patient population. However, implementation of such curricula has often been limited by inadequate resources and challenges in establishing consistent or standardized interventions.^6–9^ As a result, many health professionals and students have found their education on issues of health disparities to be insufficient. For example, 85% of primary care providers and pediatricians responding to a national survey did not feel confident in their capacity to meet their patients' social needs (Metzl and Hansen, p. 128) and felt that this impedes their ability to provide care (Metzl and Hansen, p. 128).^10,11^ Seventy-six percent of medical students polled at one institution reported that their curriculum did not adequately prepare them to address race and racialized health disparities in concrete ways.^12^

To address these deficiencies in training, medical students have sought alternative means of educating themselves and their colleagues.^13^ The objective of this report is to present one such response: a student-directed conference that sought to engage community members alongside health professionals with the objective of forming antiracist interprofessional networks meaningfully equipped to recognize and address racism in health care. To achieve this end, community members and health professional practitioners and trainees were invited to participate in a forum that would explicitly and collectively address racism in medicine, with the greatest opportunity for dialogue. The conference was advertised using social media, predominantly Facebook, as well as email listservs, flyers, and word of mouth.

The structure and content of the Racism in Medicine Conference (RiMC) 2017 comprised 16 interactive workshops (Table 1) addressing a variety of topics pertinent to racism in medicine, 2 keynote addresses from experts in the field, an interprofessional panel of 5 antiracist providers, and a concluding debrief session to process experiences of participants at the conference. Speakers were selected based on active organizations and known experts in the community, and topics were selected based on the importance to medical practice as well as the greatest potential for education.

**Table 1.** Workshops Offered at Racism in Medicine Conference 2017

Title	Theme	Workshop leadership
Healthcare Providers as Allies: Exploring Strategies and Discussing Challenges	Clinical practice	Community organization leaders
First, Do No Harm: The Consequences of Police Presence in Hospitals	Politics, law, history	Medical students
Housing, Environmental Justice, and Health	Community health	Medical student
Codeswitching to Crack the Curriculum: Incorporating Racial Justice in Medical Education	Education	Medical students
Urban Firearm Violence is Structural Violence	Community health	Faculty (surgery)
Racialized Clinical Decision Making in Medical School Curricula	Education	Medical students
Coempowering Your Community in Becoming Self-Advocates for Holistic Health	Community health	Community organization leaders
Unconscious Bias in Medical Decision Making	Clinical practice	Medical residents
Fighting Divide and Conquer Politics Through Building a Statewide Movement for Healthcare as a Human Right	Politics, law, history	Community organization medical student
Political Diagnosis: Illness Narratives as a Site for Resistance	Politics, law, history	Medical students
HIV in 2017	Community health	Medical students
		Community organization leaders
Mandatory Reporting and Racial Bias	Politics, law, history	Community organization leaders
How Social Determinants Affect Healthcare for Individuals Experiencing Homelessness	Clinical practice	Community organization leaders
Race, Medicine, Mental Health, and Manifestations	Education	Medical student
A Safe Haven for the Injured? Trauma Care at the Intersection of Healthcare, Law Enforcement, and Race	Politics, law, history	Faculty (nursing)
Immigration Status and Health	Politics, law, history	Community organization leaders

## Methods

To evaluate RiMC 2017, we developed and administered a 74-item web-based survey to all attendees. We developed the survey for evaluation of this conference and it was not previously operationalized; however, members of the research team reviewed and formally tested the survey for clarity and face validity. For racial/ethnic/ancestral background, gender identity, and sexual orientation, we asked open-ended questions to accommodate intersectional, complex, and granular identities, rather than providing categories *a priori*.^14^ The survey assessed participant perceptions of content, overall conference experience, conference impact, participant activation, and pre/postperceptions of tools and knowledge gained using five-point Likert-scale responses.

In addition to these domains, additional data were collected on potential barriers to conference attendance, including physical distance and cost of attendance. We informally reviewed all open-ended comments received for emerging patterns.

Self-reported gender identity, sexual orientation, and racial and ancestral background were summarized using treemaps rather than simple tabulation. This creates a visual summary of the diversity of conference attendees without *post hoc* classification that can lead to misclassification and exclusion.^14^ Median and interquartile range (IQR) were reported as summary statistics because the distributions of continuous demographic information were skewed and these metrics are more robust to outliers and preferred for non-normal distributions.^15^ Wilcoxon signed-rank tests on five-point Likert items were used for pre–post analyses.^16^ This study was approved by the University of Pennsylvania Institutional Review Board No. 8 (IRB 829093).

## Results

### Conference composition

**FIG. 1.**
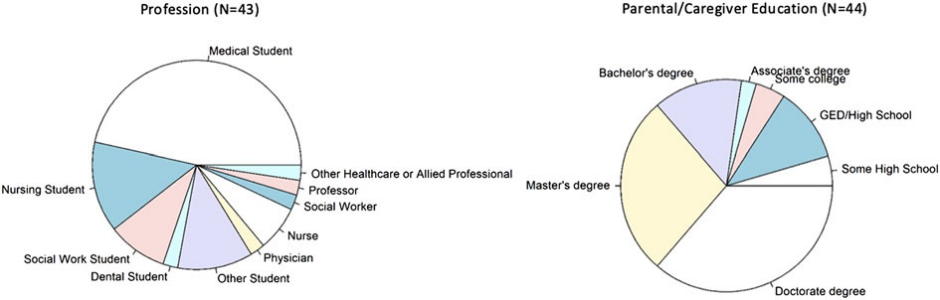
Profession and parental/caregiver educational attainment for conference attendees. Conference attendees were diverse, comprising students and health care professionals. Medical students were the largest group of attendees (45%), followed by nursing students. A majority of attendees reported parental/caregiver education beyond a bachelor's degree.

Forty-four of the 220 attendees (20%) completed the survey. Figure 1 illustrates the interprofessional diversity of the conference and also summarizes age and parent or caregiver educational attainment for respondents. Medical students were the largest group of attendees (45%), followed by nursing students (14%). Social work and dental students were in attendance, as well as students in other training programs (pre- and postbaccalaureate premedical students, public health students, and occupational therapy students). There was a smaller subset of postgraduate professionals that included physicians, social workers, nurses, and other allied health professionals such as doulas, counselors, and research scientists.

The median age of attendees was 26 (7.25) [median (IQR)], with respondents with age range between 18 and 69. We also captured information based on parent/caregiver's education attainment level. Over half of the respondents had parents with a master's or doctoral degree, with most at least having a GED or high school diploma.

**FIG. 2.**
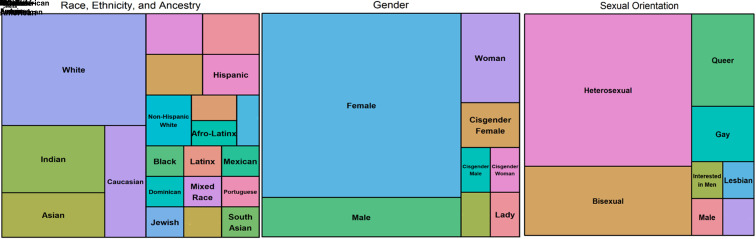
Treemaps of self-reported demographics for conference attendees (*N*=44). Racial and ethnic identities among attendees were diverse with only 34% of attendees describing themselves as white. Self-described black or African American individuals composed 7% of survey respondents and the remaining identified multiple racial, ethnic, or ancestral backgrounds. Self-reported gender was predominantly female (64%), with males representing 14% of conference attendees. Half of the respondents identified as heterosexual, with the remaining identifying as queer, bisexual, gay, or lesbian.

Racial and ethnic identities among attendees were similarly diverse (Fig. 2). Only 34% of attendees described themselves as white. Self-described black or African American individuals composed 7% of survey respondents and the remaining identified as Afro-Latinx, Asian, Asian American, Chinese American, Dominican, Hispanic, Indian, Latinx, or Mexican or responded with multiple racial, ethnic, or ancestral backgrounds, including Portuguese, Ashkenazi Jewish, and Indian, in addition to the previously mentioned groups.

Self-reported gender was predominantly female (64%), with males representing 14% (Fig. 2). Beyond these designations, individuals specifically self-identified as cisgender women, women, female/femme, and lady as their gender. Half of the respondents identified as heterosexual, with the remaining identifying as queer, bisexual, gay, or lesbian. Other responses included a repetition of gender identity and “Interested in men.”

### Baseline understanding and prior engagement

To evaluate the importance of the conference, we included questions in the survey that addressed attendee perception of the importance of understanding racism in health care as well as the presence or absence of relevant content in their prior or current training. Over 90% of attendees strongly agreed with the statement that understanding racism is important to delivering adequate health care (Table 2). Over 58% of survey respondents disagreed or strongly disagreed with the statement, “In my current or previous health professional training, I have been explicitly taught about how racism (not race) affects healthcare.”

**Table 2.** Impact of Conference Based on Pre–Post Survey Items (*N*=44)

Before/after attending the conference, to what extent do you agree with the following^a^	Pre- median (IQR)	Post- median (IQR)	*p*^b^	Respondents reporting increase %
“Understanding racism is important to delivering adequate health care.”^c^	5 (0)	5 (0)	0.174	6.98
“I am comfortable with discussing with my patients and/or colleagues how racism affects health and access to care.”	4 (0.25)	4 (1)	<0.001	47.73
“I have a generally good understanding of how racism impacts individuals' abilities to access quality care.”	4 (1)	5 (1)	<0.001	36.36
“I feel connected to other students, mentors, or colleagues who care about and are working toward understanding racism in medicine.”	4 (1)	5 (1)	<0.001	54.55
“It is important for a conference of this nature to partner with local community organizations and to support local businesses.”	5 (1)	5 (1)	0.009	22.73

^a^ All items scored 1 (strongly disagree) to 5 (strongly agree).

^b^ *p*-Value for Wilcoxon signed-rank test comparing pre- and postscores.

^c^ Results for one respondent were excluded because they did not respond to the preconference question.

### Participant perceptions of content

#### Interactive workshops

The workshops were designed and led by a diverse group of stakeholders, including medical students, community organization leaders, and health care educators. Participants were asked to evaluate the workshops with five-point Likert-scale questions, ranging from not at all satisfied to extremely satisfied regarding workshop satisfaction and not at all to entirely regarding the extent of trust in workshop leaders. Over half of the respondents reported that they entirely trusted the authority of the leaders for their respective workshops with a median of 5 (1) and 74% reported satisfaction or extreme satisfaction with their workshop [4 (1.5)] (Table 3), 76% reported that they acquired new knowledge, and 52% reported that they learned new skills (Table 3).

**Table 3.** Evaluation of Individual Conference Elements

Survey item	*N*	Median (IQR) or % yes
Interactive workshops	—	—
Scaled outcomes	—	—
To what extent did you trust your workshop leaders as authorities on their respective subjects?^a^	43	5 (1)
How satisfied were you with the workshop you attended?^b^	43	4 (1.5)
Binary outcomes	—	—
Do you feel you came away from your workshop with new knowledge?^c^	42	76.19%
Do you feel that you came away from your workshop with new practical skills?^c^	42	52.38%
Keynote: system, provider, and patient approaches to addressing inequities in health	—	—
How motivated or inspired were you by the keynote address?^d^	43	4 (2)
How relevant or important did you find the content of the keynote address?^e^	43	5 (1)
To what extent was the content of the keynote address new to you?^f^	43	3 (1.5)
Keynote: the problem of racial stratification in human health	—	—
How motivated or inspired were you by the keynote address?^d^	41	5 (1)
How relevant or important did you find the content of the keynote address?^e^	41	5 (1)
To what extent was the content of the keynote address new to you?^f^	41	4 (1)
Panel: Existing Within and Challenging Racist Structures in Medicine	—	—
How satisfied were you with the panel?^b^	42	4 (1)
How relevant or important did you find the content of the panel to the Racism in Medicine Conference?^e^	41	4 (2)
The panel represented a diverse range of voices, backgrounds, and professions relevant to the panel topic^g^	40	4 (2)
Debrief sessions		
How valuable did you find the debrief sessions?^h^	26	4 (1)

^a^ Scored 1 (not at all) to 5 (entirely).

^b^ Scored 1 (not at all satisfied) to 5 (extremely satisfied).

^c^ Scored yes or no.

^d^ Scored 1 (not at all motivated or inspired) to 5 (extremely motivated or inspired).

^e^ Scored 1 (not at all relevant or important) to 5 (entirely relevant or important).

^f^ Scored 1 (not at all new) to 5 (entirely new).

^g^ Scored 1 (strongly disagree) to 5 (strongly agree).

^h^ Scored 1 (not at all valuable) to 5 (extremely valuable).

IQR, interquartile range.

#### Keynotes

Respondents were asked with five-point Likert-scale questions to rank the importance and novelty of the content of the two keynote addresses, from not at all relevant or important to entirely relevant or important and not at all new to entirely new, as well as rank how motivating or inspiring they found each address, ranging from not at all motivated or inspired to extremely motivated or inspired.

For the first keynote, 65% of respondents reported some degree of motivation or inspiration with a median score of 4 (2), 86% felt the keynote was relevant and important with a median score of 5 (1), and 35% felt they learned new content with a median score of 3 (1.5) for how new the content was to them (Table 3). For the second keynote, 83% of respondents reported motivation or inspiration with a median score of 5 (1), 93% found the content to be important and relevant with a median score of 5 (1), and 61% felt they learned new information with a median score of 4 (1) (Table 3).

#### Panel presentations

The panel on existing within and challenging racist structures in medicine included representation from social work, nursing, medical residents and physicians, faculty, and students and was assembled to also be gender and racially inclusive. With five-point Likert-scale questions, survey respondents were asked to evaluate the relevance/importance of the conference (ranging from not at all relevant or important to entirely relevant or important), the diversity of perspectives on the panel (ranging from strongly disagree to strongly agree) in scaled responses, as well as their general satisfaction (ranging from not at all satisfied to extremely satisfied). The median respondent score was 4 across all three metrics; perception of the panel as important/relevant (1), panel diversity (2), and satisfaction (2), indicating a positive reception of the panel by the majority of attendees (Table 3).

#### Debriefing sessions

There was significant decline in attendance before the closing debriefing session, and this is reflected in the decrease in number of responses to the question (five-point Likert scale, ranging from not at all valuable to extremely valuable) about this aspect of the conference (*N*=26). Among these respondents, the median score for the value of the sessions was 4 (1) and 65% felt that debriefing was valuable or extremely valuable (Table 3).

### Overall conference experience

We asked survey respondents to evaluate the information and practical skills provided by the conference in a five-point scale response (ranging from strongly disagree to strongly agree), The median response score for new information was 5 (1) and for new practical skills was 3 (1) (Table 4). They were also asked to rank from 1 to 5 (strongly disagree to strongly agree) how effectively the conference organizers partnered with community and supported local businesses. The median response score was 4 (1) when asked if RiMC effectively partnered with community organizations on conference content (Likert score 4 or 5) and 4 (1) reported that they felt RiMC effectively supported local businesses (Table 4).

**Table 4.** Evaluation of Overall Conference Experience and Impact

Survey item	*N*	Median (IQR)
This conference provided me with information that was not covered in my current or previous educational experiences.^a^	43	5 (1)
This conference provided me with practical skills that I had not obtained through my current or previous educational experiences.^a^	43	3 (1)
How well do you feel RiMC did in partnering with community organizations for conference content?^b^	42	4 (1)
How well do you feel RiMC did in supporting local businesses and organizations?^b^	42	4 (1)
This conference provided me with the motivation and energy to combat racism in medicine.^a^	43	4 (1)
This conference provided me with a network of individuals with whom I can work toward combating racism in medicine.^a^	43	4 (1)
Do you plan to disseminate things you learned at RiMC informally at your home institution or workplace (peer-to-peer communication)?^c^	42	5 (1)
Do you plan to disseminate things you learned at RiMC formally at your home institution or workplace (planned events)?^c^	42	4 (2)

^a^ Scored 1 (strongly disagree) to 5 (strongly agree).

^b^ Scored 1 (not at all well) to 5 (very well).

^c^ Scored 1 (very unlikely) to 5 (certainly).

RiMC, Racism in Medicine Conference.

### Conference impact and participant activation

We assessed with five-point scale questions, ranging from strongly disagree to strongly agree, whether survey respondents felt that the conference motivated or energized them and provided them with a network of individuals similarly motivated. The median response score was 4 (1) for the conference providing motivation or energy to combat racism and 4 (1) for providing a network of individuals to work with toward combating racism in medicine (Table 4). Additionally, we assessed how likely respondents were to share information attained at the conference informally and formally, with two five-point Likert-scale questions ranging from very unlikely to certainly. On average, survey respondents scored their likelihood to informally disseminate the information at their home institution through peer to peer at a median of 5 (1) and likelihood to do so formally through planned events as 4 (2) (Table 4).

### Pre/postperceptions of skills and knowledge gained

To quantify the impact of the conference on participants, the survey posed a series of five-point scale questions (ranging from strongly disagree to strongly agree) that assessed their pre- and postconference understanding and engagement of racism in medicine. Several of the questions included in the pre–post analysis overlap with those discussed above and included in Table 4, but differ by allowing us to more directly measure change related to conference attendance.

There were statistically significant increases between pre- and postresponse scores for the degree of comfort discussing with patients and/or colleagues about how racism affects health and access to care (*p*<0.001), understanding of how racism impacts individuals' abilities to access quality care (*p*<0.001), and feeling connected to other students, mentors, or colleagues who care about and are working toward understanding racism in medicine (*p*<0.001, Table 2). The largest increase was in connection to other students, mentors, or colleagues, suggesting that the conference was addressing an unmet need of connecting with similarly conscious individuals working to understanding racism in medicine. Additionally, 47% of respondents reported exchanging contact with other individuals at the conference.

There was also a statistically significant increase in response scores for the importance of conferences of this nature to partner with local community organizations and businesses (*p*=0.009). This relates to the explicit goal of the conference to partner with minority-owned businesses and community organizations committed to racial justice. At baseline, the median score was 4 (1), suggesting that this was already a value held by attendees, but the increase shows that the conference emphasis on this was perceived and appreciated. In the pre–post analysis, one item—understanding racism is important to delivering adequate health care—did not show a statistically significant increase, likely due to the high baseline score [pre: 5 (0), post: 5 (0)] (Table 2).

### Assessing potential barriers

We also assessed potential barriers to this conference and its future iterations by collecting information on the distance individuals traveled to attend and how much respondents would have been willing to pay for the conference if it had not been offered free of charge. Over two-thirds of respondents came from within 10 miles of the host institution, but 14% respondents traveled >100 miles, with the remaining coming from intermediate distances. Nearly half (48%) of the respondents reported they would be willing to pay between $10 and $20 to attend the conference, and another 27% reported they would be willing to pay between $20 and $50 for the conference.

## Discussion

RiMC 2017 was a student-driven educational intervention that sought to address health and allied professionals' lack of training in racism and its impact on health care. Over half of the RiMC 2017 attendees perceived their prior training as lacking in vital content that this conference was designed to provide. As a school administrator commented, racism in medicine “has often been taught in a decontextualized manner, offering no historical or social backdrop … this is dangerous because it can predispose people to thinking it has to do with biological differences, whereas race is a social construct … and if you don't understand the context you don't truly understand the problem.” Attendees came from diverse racial and ethnic backgrounds, representing a broad spectrum of gender identities and inclusive of sexual minorities.

The conference expanded on the collaborative structure of previous iterations, featuring intra- and interinstitutional cosponsorship from three health professional schools and five additional medical schools in the Greater Philadelphia area. Our results suggest that the conference succeeded in building a broadly collaborative, informative, inspiring, interprofessional educational experience for health and allied professionals and trainees from disciplines such as medicine, dentistry, social work, nursing, community organizing, and activism.

Survey responses indicated that attendees intend to propagate the content from RiMC at their home institutions. As an activist working in a health care setting said, “I did social media blasts the entire time I was at the conference … I looped the head of diversity at [their home institution]… it was helpful to have educational conversations with people who already knew the terminology.” The importance of interdisciplinary and community participation to the success of the conference suggests a necessity for a pedagogical shift. Rather than elevating the unilateral perspective of physician experts, this conference employed the expertise of allied health professionals, students, and importantly, community members and activists to teach the importance of structural humility, ways in which structural forces affect patients, and best practices for combating these injustices.

While the conference was generally successful at meeting its goals, there are several limitations worth mentioning. Although the conference addressed a wide range of content, survey respondents identified gaps in conference programming: the intersection of racism with women and children's health, practical skills training for addressing colleagues' racist practices, and workshops focused on patient empowerment. Furthermore, although the conference planning committee liaised with students from other medical and allied professional schools, the relationships consisted mainly of financial and logistical collaborations rather than true interdisciplinary conference content design. Future organizers may consider working more closely with partnering institutions in the conference's planning to better foster interinstitutional relationships and further diversify conference content.

In addition, attendees to the conference were self-selected and, as such, appeared largely informed about the broader mission of addressing racism. Specifically, one might assume that those individuals motivated to attend this conference may already have a high baseline knowledge of the covered content and therefore could perceive less benefit compared with peers less motivated to attend and, by the same logic, less informed in those content areas. However, because of their potential bias, the attendees may also be primed to gain more from the conference. It is not possible to determine if either of these effects is present or which might dominate in the current study. Therefore, future interventions in populations with less initial buy-in should be evaluated for comparison.

The small number of submitted comments limited the scope of our qualitative assessment. A comprehensive qualitative analysis would be useful to understand the nuances about attendees' prior experiences, what led to them attending the conference, and what they learned. Future evaluations of similarly structured conferences should utilize either semistructured interviews or more open-ended survey questions designed to elicit comments to collect such qualitative data. Similarly, methodologies such as focus groups could be conducted for further qualitative analysis. Finally, the response rate for the conference survey was 20%; however, this is consistent with prior online response rates in educational settings.^17^ In future conferences, we foresee utility in collecting demographic data on all participants to determine if survey respondents are representative of attendees overall.

## Conclusion

RiMC 2017 was a student-directed, community-engaged, interprofessional learning opportunity addressing how racism impacts health care access and contributes to health inequity. This conference provided knowledge and skills relevant to the antiracist practice of medicine and facilitated connections between stakeholders committed to improving health in marginalized racial minority communities. Future work should focus on enhancing community and interschool partnerships, providing more skill-based training, and carrying out more robust analyses of the impact to facilitate sustainable replication and expansion of this conference and similar educational interventions at other institutions in future years.

## Abbreviations Used

IQR: interquartile range
LCME: Liaison Committee on Medical Education
OID: Office of Inclusion Diversity
PDI: Program for Diversity and Inclusion
RiMC: Racism in Medicine Conference
